# Osseous Sarcoidosis Mimicking Metastatic Disease in a Patient With Longstanding Pulmonary Sarcoidosis: A Case Report

**DOI:** 10.7759/cureus.101402

**Published:** 2026-01-12

**Authors:** Syed M Naqvi, Iftekhar Bader, Haylee Eliasson

**Affiliations:** 1 Pulmonary Medicine, Chicago Medical School/Rosalind Franklin University of Medicine and Science, North Chicago, USA; 2 Pulmonary Medicine, Aurora Medical Center, Kenosha, USA

**Keywords:** multisystem sarcoidosis, non-necrotizing granuloma, osseous sarcoidosis, pulmonary sarcoidosis, sarcoidosis

## Abstract

Sarcoidosis is a multisystem granulomatous disease in which osseous involvement is uncommon and most often affects the small bones of the hands and feet, whereas axial skeletal disease remains underrecognized and may mimic metastatic malignancy. A 72-year-old man with longstanding pulmonary sarcoidosis and moderate persistent asthma was incidentally found to have multifocal sacral and iliac bone lesions on lumbar spine magnetic resonance imaging (MRI) obtained for evaluation of back pain and radiculopathy. These lesions were radiologically suspicious for osseous metastases, prompting systemic staging with fluorodeoxyglucose positron emission tomography-computed tomography (FDG PET-CT) and referral to hematology/oncology. PET-CT demonstrated low-level FDG uptake within the sacral and iliac lesions, diffuse splenic hypermetabolism, and calcified mediastinal and hilar lymph nodes compatible with prior granulomatous disease, but no hypermetabolic lymphadenopathy or dominant osseous mass. CT-guided biopsy of a left iliac crest lesion revealed focal non-necrotizing granulomas negative for acid-fast bacilli, fungal organisms, and malignancy, establishing the diagnosis of osseous sarcoidosis in the context of systemic disease. The patient’s back pain improved with conservative measures, and he remained without progressive pulmonary or skeletal symptoms under multidisciplinary surveillance. This case highlights that vertebral and pelvic bone lesions in older patients with granulomatous lung disease may represent sarcoidosis rather than metastatic cancer and underscores the importance of tissue confirmation when imaging findings are indeterminate.

## Introduction

Sarcoidosis is an inflammatory multisystem disease of unknown etiology characterized by non-necrotizing granulomas and most commonly involves the lungs and intrathoracic lymph nodes [1,2]. Osseous sarcoidosis occurs in approximately 3-13% of patients and was historically thought to predominantly affect the small bones of the hands and feet [3]. Sarcoidosis frequently presents as a multisystem disease with heterogeneous clinical phenotypes and variable organ involvement [4]. With the increasing use of magnetic resonance imaging (MRI) and fluorodeoxyglucose positron emission tomography-computed tomography (FDG PET-CT), axial skeletal involvement, particularly of the spine and pelvis, has been increasingly recognized among patients with bone sarcoidosis [3].

Radiologic findings of spinal and pelvic sarcoid lesions are nonspecific and frequently mimic metastatic carcinoma, multiple myeloma, lymphoma, and infectious osteomyelitis [5]. Although MRI is highly sensitive for detecting marrow abnormalities, it cannot reliably differentiate sarcoidosis from metastatic disease based on signal characteristics alone [6]. FDG PET-CT is similarly sensitive for detecting osseous involvement but lacks specificity, as both inflammatory granulomatous disease and malignancy may demonstrate increased tracer uptake [7]. Consequently, histopathologic confirmation is often required, particularly in older patients with competing oncologic risk factors, to avoid misclassification as metastatic disease and inappropriate treatment [8].

## Case presentation

A 72-year-old man with a history of radiographically established pulmonary sarcoidosis, moderate persistent asthma, coronary artery disease, hypertension, hyperlipidemia, benign prostatic hyperplasia, nephrolithiasis, and gastroesophageal reflux disease was seen in the pulmonary clinic for routine follow-up. He was a lifelong never-smoker, consumed alcohol rarely, and denied occupational or environmental exposures to silica, metal dusts, or agricultural chemicals. There was no family history of granulomatous or autoimmune disease.

**Figure 1:**
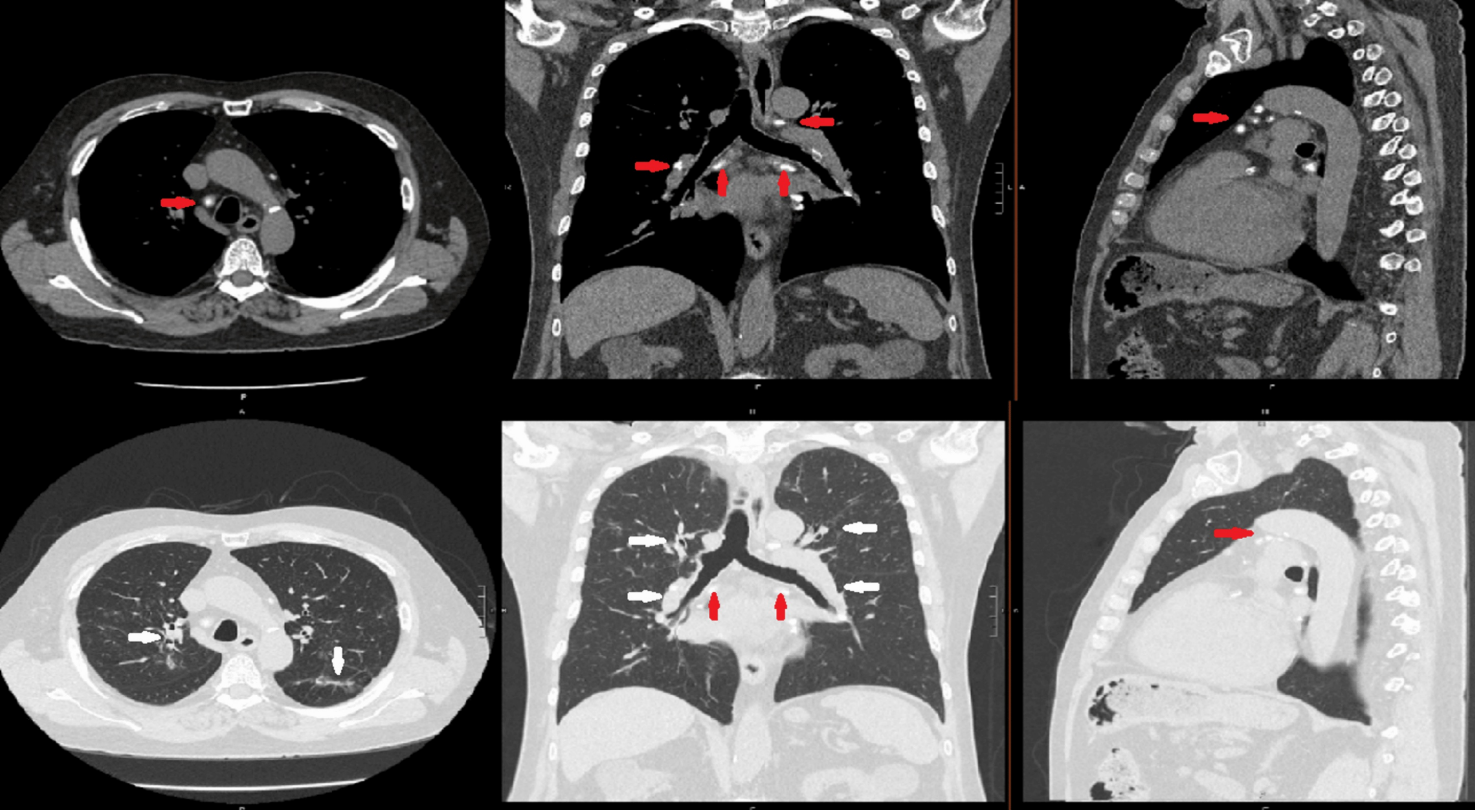
Axial, sagittal, and coronal chest computed tomography images, in mediastinal and lung windows, demonstrating stable calcified mediastinal and hilar lymphadenopathy, marked by red arrows, and persistent, mostly perihilar and perilymphatic nodular disease, marked by white arrows.

Pulmonary sarcoidosis had been recognized several years earlier following cardiac CT calcium scoring and subsequent dedicated chest imaging, which demonstrated calcified mediastinal and hilar lymphadenopathy with perilymphatic micronodular opacities in a peribronchovascular and subpleural distribution. Serial chest CT scans from 2021 through 2025 showed stable calcified mediastinal and hilar lymphadenopathy, persistent perihilar perilymphatic nodular disease, moderate air trapping, and chronic partial collapse of the right middle lobe without suspicious pulmonary masses or progressive parenchymal fibrosis. The most recent chest CT scan images are shown in Figure 1.

Bronchoscopy with bronchoalveolar lavage and right upper lobe transbronchial biopsy performed in 2021 demonstrated benign respiratory epithelium and pulmonary histiocytes without granulomas, vasculitis, malignancy, or pathogenic organisms. Given the characteristic thoracic imaging findings and chronic respiratory symptoms in a never-smoker, the overall clinical picture was considered most consistent with pulmonary sarcoidosis. The patient had never received systemic corticosteroid therapy for sarcoidosis and was managed with inhaled triple therapy, montelukast, and as-needed short-acting beta-agonist for asthma. Pulmonary function testing over several years demonstrated a stable moderate obstructive ventilatory defect with air trapping and low-normal diffusing capacity, without restrictive physiology.

**Figure 2:**
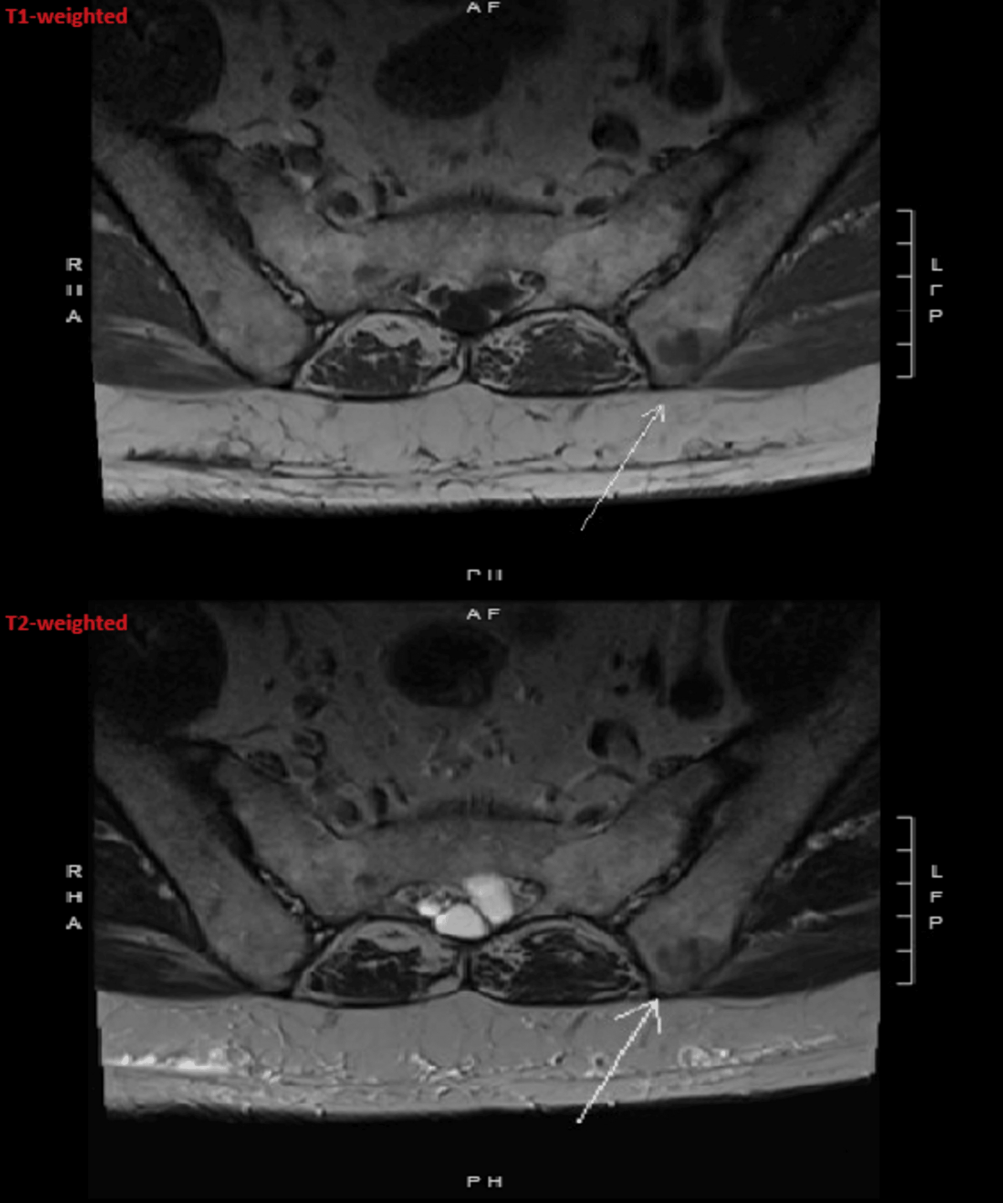
Magnetic resonance imaging of the lumbar spine without contrast demonstrating a lesion with low signal intensity on both T1-weighted and T2-weighted sequences involving the iliac bone (marked by the white arrow).

Approximately four years after the initial recognition of pulmonary sarcoidosis, the patient developed new lower back pain with radicular symptoms radiating into the lower extremity and was evaluated by orthopedics. MRI of the lumbar spine without contrast demonstrated multiple lesions with low signal intensity on both T1-weighted and T2-weighted sequences involving the sacrum, iliac bones, and the L4 vertebral body. These lesions were interpreted as concerning for osseous metastases, prompting further evaluation (Figure 2).

**Figure 3:**
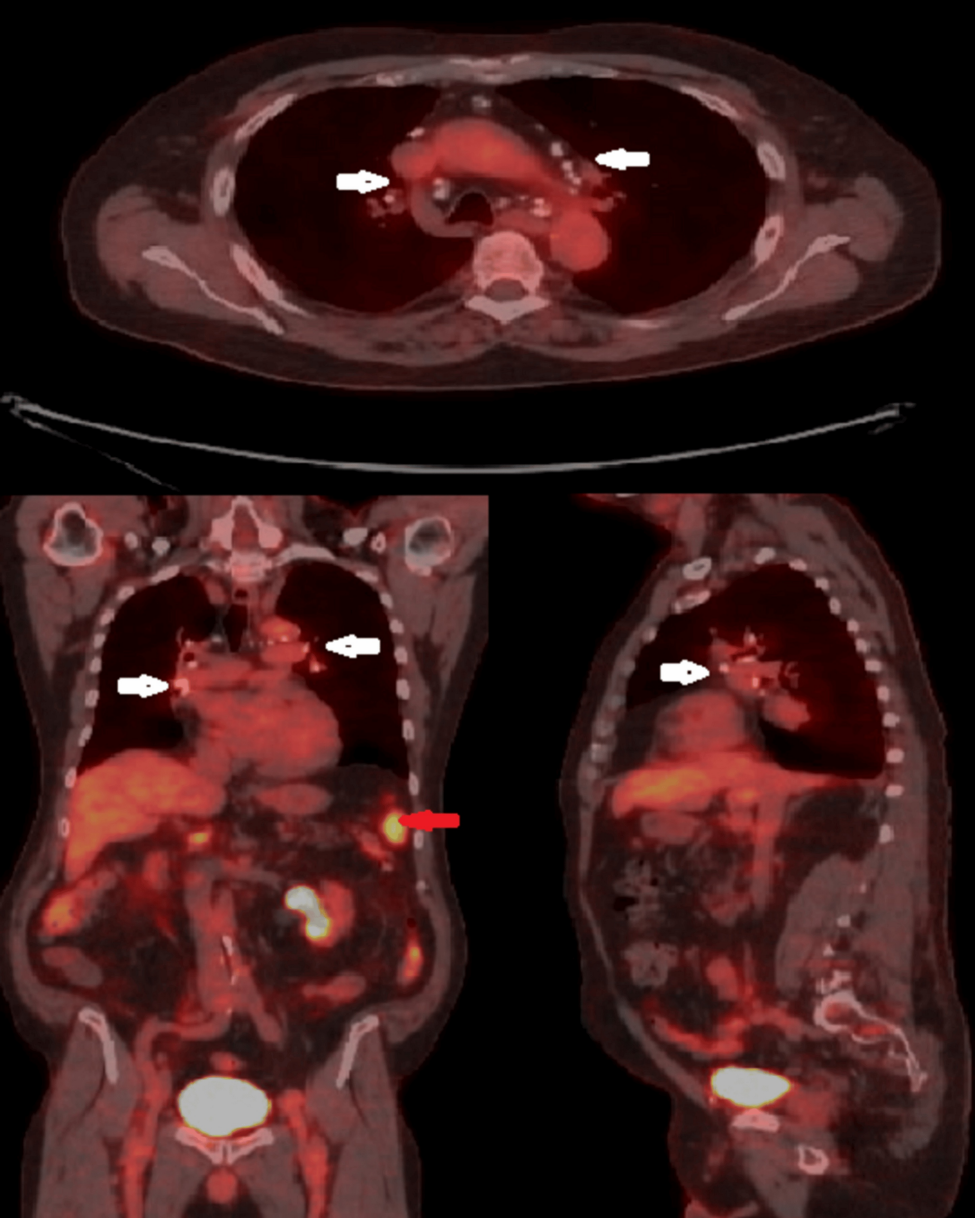
Positron emission tomography-computed tomography demonstrating symmetric calcified mediastinal and bilateral hilar lymph nodes, marked by white arrows, and splenic involvement with diffuse increased fluorodeoxyglucose uptake, marked by red arrow.

FDG PET-CT demonstrated no hypermetabolic pulmonary masses or lymphadenopathy, with stable symmetric calcified mediastinal and bilateral hilar lymph nodes consistent with inactive granulomatous lung disease, the spleen was normal in size and demonstrated diffuse increased FDG uptake with a maximum standardized uptake value of approximately 9.5 suggestive of splenic involvement, and osseous abnormalities involving the sacrum, iliac bones, and lumbar vertebrae demonstrated low-level FDG uptake comparable to background marrow activity without evidence of intensely FDG-avid or aggressive vertebral lesions (Figure 3).

Given the patient’s age and multifocal axial skeletal lesions, he was referred to hematology and oncology for further evaluation. Laboratory studies, including complete blood count, comprehensive metabolic panel, serum and urine protein electrophoresis, and plasma cell dyscrasia workup, were unremarkable. Prostate-specific antigen levels were mildly elevated but stable for the last three years and were already under active surveillance by urology, with no clinical or radiographic evidence of metastatic prostate cancer. Detailed laboratory results are summarized in Table 1.

**Table 1:** Laboratory investigations.

Parameter	Patient value	Reference range
Complete blood count		
Hemoglobin	13.1 g/dL	13.5–17.5 g/dL
Hematocrit	39.4%	41–53%
White blood cell count	4.5 × 10³/µL	4.0–11.0 × 10³/µL
Platelet count	175 × 10³/µL	150–400 × 10³/µL
Comprehensive metabolic panel		
Sodium	143 mmol/L	135–145 mmol/L
Potassium	4.3 mmol/L	3.5–5.1 mmol/L
Chloride	107 mmol/L	98–107 mmol/L
Bicarbonate (CO₂)	27 mmol/L	22–29 mmol/L
Blood urea nitrogen	22 mg/dL	7–20 mg/dL
Creatinine	1.02 mg/dL	0.6–1.3 mg/dL
Estimated glomerular filtration rate	78 mL/minute/1.73 m²	≥60 mL/minute/1.73 m²
Glucose	110 mg/dL	70–99 mg/dL
Calcium	9.2 mg/dL	8.6–10.2 mg/dL
Liver function and proteins		
Total bilirubin	0.8 mg/dL	0.2–1.2 mg/dL
Aspartate aminotransferase	18 U/L	10–40 U/L
Alanine aminotransferase	31 U/L	7–56 U/L
Alkaline phosphatase	64 U/L	44–147 U/L
Albumin	3.8 g/dL	3.5–5.0 g/dL
Total protein	6.9 g/dL	6.0–8.3 g/dL
Sarcoidosis-related and inflammatory markers		
Angiotensin-converting enzyme	48 U/L	9–67 U/L
Lactate dehydrogenase	179 U/L	140–280 U/L
Immunoglobulin G	957 mg/dL	700–1600 mg/dL
Iron storage and vitamins		
Ferritin	22 ng/mL	30–400 ng/mL
Vitamin B12	536 pg/mL	200–900 pg/mL
Folate	10.9 ng/mL	≥4.0 ng/mL
25-hydroxyvitamin D	53.0 ng/mL	30–100 ng/mL
Endocrine and coagulation		
Thyroid-stimulating hormone	2.519 µIU/mL	0.4–4.5 µIU/mL
Hemoglobin A1c	5.4%	4.0–5.6%
Prothrombin time	10.7 seconds	11–13.5 seconds
International normalized ratio	1.1	0.8–1.1
Prostate-specific antigen	7.62 ng/mL	≤4.0 ng/mL
Serum immunoglobulins and light chains		
Immunoglobulin A	173 mg/dL	70–400 mg/dL
Immunoglobulin M	157 mg/dL	40–230 mg/dL
Kappa free light chain	3.05 mg/dL	0.33–1.94 mg/dL
Lambda free light chain	1.70 mg/dL	0.57–2.63 mg/dL
Kappa to lambda ratio	1.79	0.26–1.65
Urinalysis		
Color	Yellow	Yellow
Clarity	Clear	Clear
Specific gravity	1.015	1.005–1.030
pH	6.0	5.0–8.0
Glucose	Negative	Negative
Ketones	Negative	Negative
Protein	Negative	Negative
Blood	Negative	Negative
Leukocyte esterase	Trace	Negative
Nitrite	Negative	Negative
Bilirubin	Negative	Negative
Urobilinogen	0.2	0.1–1.0
Red blood cells	None	0–2 per HPF
White blood cells	None	0–5 per HPF
Squamous epithelial cells	1 to 5	0–5 per HPF
Bacteria	None seen	None seen
Calcium oxalate crystals	Moderate	None
Uric acid crystals	Few	None

**Figure 4:**
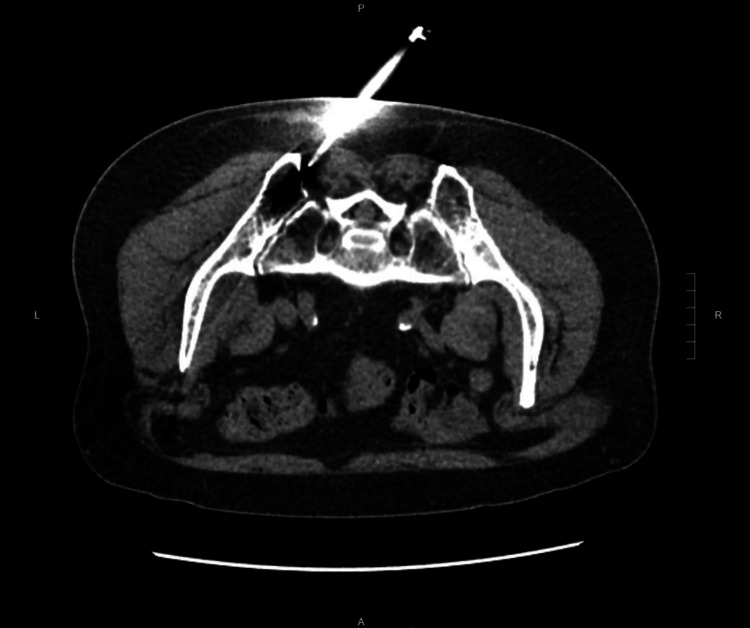
Computed tomography-guided core biopsy of a left iliac crest lesion.

To establish a definitive diagnosis, a CT-guided core biopsy of a left iliac crest lesion was performed (Figure 4).

**Figure 5:**
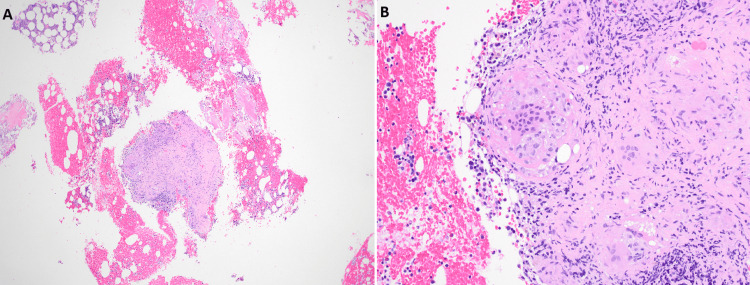
Microscopy of computed tomography-guided core biopsy of a left iliac crest lesion showing focal non-necrotizing granulomas composed of epithelioid histiocytes and multinucleated giant cells. Photomicrographs “A” and “B” are shown at low magnification (4×) and high magnification (20×), respectively.

Histopathologic examination demonstrated focal non-necrotizing granulomas composed of epithelioid histiocytes and multinucleated giant cells (Figure 5). Special stains for acid-fast bacilli and fungal organisms were negative, and no evidence of malignancy was identified.

These findings, in the context of established systemic granulomatous disease, confirmed the diagnosis of osseous sarcoidosis involving the sacrum and iliac bones.

Following the biopsy, the patient reported significant improvement in his lower back pain with conservative management, including chiropractic therapy. He denied focal bone pain, neurologic deficits, gait disturbance, constitutional symptoms, or new musculoskeletal complaints. His respiratory status remained stable, with exertional dyspnea that had slowly progressed over several years but without acute exacerbations, resting hypoxemia, or functional limitation beyond reduced walking pace.

Given his clinical improvement, absence of neurologic compromise or structural instability, and stable pulmonary disease, systemic immunosuppressive therapy was not initiated. The patient continued inhaled therapy for asthma and remained under multidisciplinary surveillance, with planned interval chest imaging, skeletal imaging, and annual pulmonary function testing. He continued established follow-up with cardiology, including routine electrocardiographic and cardiac imaging surveillance as previously arranged, and ongoing annual ophthalmologic examinations for monitoring of ocular involvement. A rheumatology consultation was arranged to reassess the need for systemic therapy should skeletal or other systemic disease progression occur.

## Discussion

Osseous sarcoidosis is an uncommon but clinically important manifestation of systemic sarcoidosis that may be underrecognized, particularly when it involves the axial skeleton and radiographically mimics metastatic disease [3]. Earlier series reported bone involvement in 3-13% of sarcoidosis patients and suggested a predilection for the small bones of the hands and feet [5].

In a large single-center cohort, Zhou et al. demonstrated that axial skeletal involvement, including the spine and pelvis, is common among patients with bone sarcoidosis and is frequently associated with multi-organ disease [3]. The diffuse splenic FDG uptake and chronic thoracic lymphadenopathy observed in the present case are consistent with this phenotype of multisystem sarcoidosis.

MRI is highly sensitive for detecting osseous sarcoid lesions but cannot reliably differentiate sarcoidosis from metastatic disease based on marrow signal characteristics alone [6]. FDG PET-CT similarly detects metabolically active lesions but lacks specificity, as both inflammatory granulomatous disease and malignancy may demonstrate increased tracer uptake [7]. As a result, imaging findings alone are insufficient to establish a definitive diagnosis in many patients.

Bone or bone marrow biopsy demonstrating non-necrotizing granulomas in the appropriate clinical context remains the diagnostic gold standard for osseous sarcoidosis [8]. Establishing the correct diagnosis is essential, as management strategies differ substantially between sarcoidosis and metastatic malignancy, and misclassification may lead to unnecessary oncologic therapy.

There is no universally accepted treatment algorithm for bone sarcoidosis, and management is individualized based on symptom burden, lesion location, and overall disease activity [9]. Systemic corticosteroids remain first-line therapy for symptomatic disease, with antimetabolites and biologic agents reserved for refractory cases [10]. Long-term management should also consider bone health, particularly in patients exposed to corticosteroids or with osseous involvement [11].

Tumor necrosis factor-alpha inhibitors, including infliximab and adalimumab, have demonstrated efficacy in refractory osseous sarcoidosis involving the axial skeleton [12,13]. Case reports have also described successful treatment with adalimumab in patients intolerant of or refractory to other therapies [14].

In the present case, conservative management with close surveillance was appropriate given symptomatic improvement, absence of neurologic compromise, and stable pulmonary disease. Long-term follow-up remains essential, as osseous sarcoidosis may reflect a chronic multisystem disease course with potential for progression [9].

## Conclusions

Axial osseous sarcoidosis is an underrecognized manifestation of systemic sarcoidosis that can closely mimic metastatic malignancy on advanced imaging. MRI and FDG PET-CT are highly sensitive for detecting osseous lesions but lack sufficient specificity to reliably distinguish granulomatous inflammation from malignancy. In patients with known or suspected granulomatous lung disease, tissue confirmation is therefore essential when imaging findings are indeterminate to avoid misdiagnosis and inappropriate oncologic treatment. This case highlights the critical role of biopsy in establishing the diagnosis of osseous sarcoidosis and demonstrates that asymptomatic or minimally symptomatic axial skeletal involvement may follow an indolent course amenable to conservative management and close surveillance. Increased clinical awareness of axial osseous sarcoidosis, along with careful integration of imaging findings, histopathology, and clinical context, is essential to ensure accurate diagnosis and individualized management strategies.
